# Global mtDNA genetic structure and hypothesized invasion history of a major pest of citrus, *Diaphorina citri* (Hemiptera: Liviidae)

**DOI:** 10.1002/ece3.3680

**Published:** 2017-11-26

**Authors:** Yufa Luo, Ingi Agnarsson

**Affiliations:** ^1^ School of Life and Environmental Sciences Gannan Normal University Ganzhou China; ^2^ Department of Biology University of Vermont Burlington VT USA; ^3^ Department of Entomology National Museum of Natural History Washington DC USA

**Keywords:** Asian citrus psyllid, biological control, global genetic structure, invasion history, phylogeography

## Abstract

The Asian citrus psyllid *Diaphorina citri* Kuwayama is a key pest of citrus as the vector of the bacterium causing the “huanglongbing” disease (HLB). To assess the global mtDNA population genetic structure, and possible dispersal history of the pest, we investigated genetic variation at the *COI* gene collating newly collected samples with all previously published data. Our dataset consists of 356 colonies from 106 geographic sites worldwide. High haplotype diversity (H‐mean = 0.702 ± 0.017), low nucleotide diversity (π‐mean = 0.003), and significant positive selection (Ka/Ks = 32.92) were observed. Forty‐four haplotypes (Hap) were identified, clustered into two matrilines: Both occur in southeastern and southern Asia, North and South America, and Africa; lineages A and B also occur in eastern and western Asia, respectively. The most abundant haplotypes were Hap4 in lineage A (35.67%), and Hap9 in lineage B (41.29%). The haplotype network identified them as the ancestral haplotypes within their respective lineages. Analysis of molecular variance showed significant genetic structure (*F*_ST_ = 0.62, *p* < .0001) between the lineages, and population genetic analysis suggests geographic structuring. We hypothesize a southern and/or southeastern Asia origin, three dispersal routes, and parallel expansions of two lineages. The hypothesized first route involved the expansion of lineage B from southern Asia into North America via West Asia. The second, the expansion of some lineage A individuals from Southeast Asia into East Asia, and the third involved both lineages from Southeast Asia spreading westward into Africa and subsequently into South America. To test these hypotheses and gain a deeper understanding of the global history of *D. citri*, more data‐rich approaches will be necessary from the ample toolkit of next‐generation sequencing (NGS). However, this study may serve to guide such sampling and in the development of biological control programs against the global pest *D. citri*.

## 1 INTRODUCTION

Genetic structure is the nonrandom distribution of alleles or genotypes in space or time (Mahy, Vekemans, & Jacquemart, 1999). Studies on population genetic structure can estimate genetic diversity and gene flow among populations, infer ancestral populations, and population level phylogeography. Such information is essential for informed management of pest species (Porretta, Canestrelli, Bellini, Celli, & Urbanelli, 2007). In recent years, understanding of biological invasion mechanism has increasingly relied on knowledge of the genetic structure of invasive species (Lee, 2002). Such studies can help to illuminate the basic biology and ecology of invasive species, the relationships between the intrinsic genetic characteristics of biological invaders, and their successful invasions (Xu, Zhang, Lu, & Chen, 2001). Furthermore, they can aid in the identification of effective biological control agents (Hoy, 2013).

**Figure 1:**
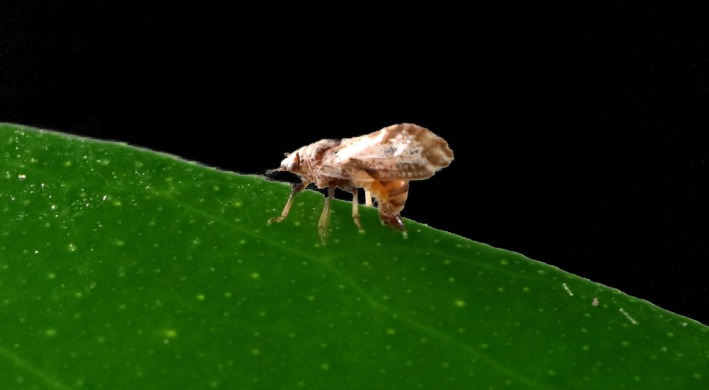
A photograph of *Diaphorina citri* Kuwayama female adult

The Asian citrus psyllid *Diaphorina citri* Kuwayama (Hemiptera: Liviidae) (Figure 1) is a world‐wild economic pest because it transmits the bacterial pathogen, *Candidatus* Liberibacter, that causes citrus greening disease (huanglongbing, HLB), considered one of the most serious diseases of citrus (da Graça, 2008). The psyllid was first described from Taiwan in 1907 (Halbert & Manjunath, 2004). However, the available evidence suggests that *D. citri* originates from the Indian subcontinent (Hollis, 1987) and has subsequently expanded to other subtropical and tropical regions of Asia and also to the Indian Ocean islands of Réunion and Mauritius (Waterhouse, 1998), and South America (Halbert & Núñez, 2004). Recently, it has reached Central American countries and southern USA (Halbert & Manjunath, 2004).

Environmental factors, such as temperature, humidity, host plants, and human activities, influence the survival and reproduction of *D. citri* (Wang, 2002; Waterhouse, 1998; Xu, Xia, & Ke, 1994; Yang, 1989; Zhao, 1987). However, *D. citri* is characterized by high reproductive output and rapid generation turnover (2–7 weeks) and may thus rapidly evolve in response to new environments after an invasion. If semi‐isolated subpopulations acquire different ecological traits, such as in host preference, development time, pathogenicity, vector capacity, susceptibility to pesticides, and tolerance to heat, and other abiotic stressors (Carmichael et al., 2007; Jourdie et al., 2010; Pinho, Harris, & Ferrand, 2007; Remais, Xiao, Akullian, Qiu, & Blair, 2011), then genetically divergence among these subpopulations may rapidly emerge. Genetic studies have the potential to trace the *D. citri* demographic history that determines population genetic diversity of the invasive species and thus to illuminate geographic origin and secondary translocation events. However, to date, the global genetic differentiation, phylogeographic relationships, and invasion history were not resolved mainly owing limited geographic scope and relatively small sample sizes of prior studies (Boykin et al., 2012; de León et al., 2011; Guidolin, Fresia, & Cônsoli, 2014; Lashkari et al., 2014).

To assess the mtDNA population genetic structure, and potential dispersal routes of *D. citri*, we study global mtDNA genetic differentiation and phylogeography. We sequenced partial fragments of mitochondrial *COI* gene of recently collected individuals invasive to China and augmented our sampling with sequences from GenBank covering nearly the entire geographic range of *D. citri*. The current dataset covers 106 distinct localities around the globe. It thus offers a more holistic insight into *D. citri* matriline history than have prior studies.

## 2 MATERIALS AND METHODS

### 2.1 Insect collection and sequencing

**Figure 2:**
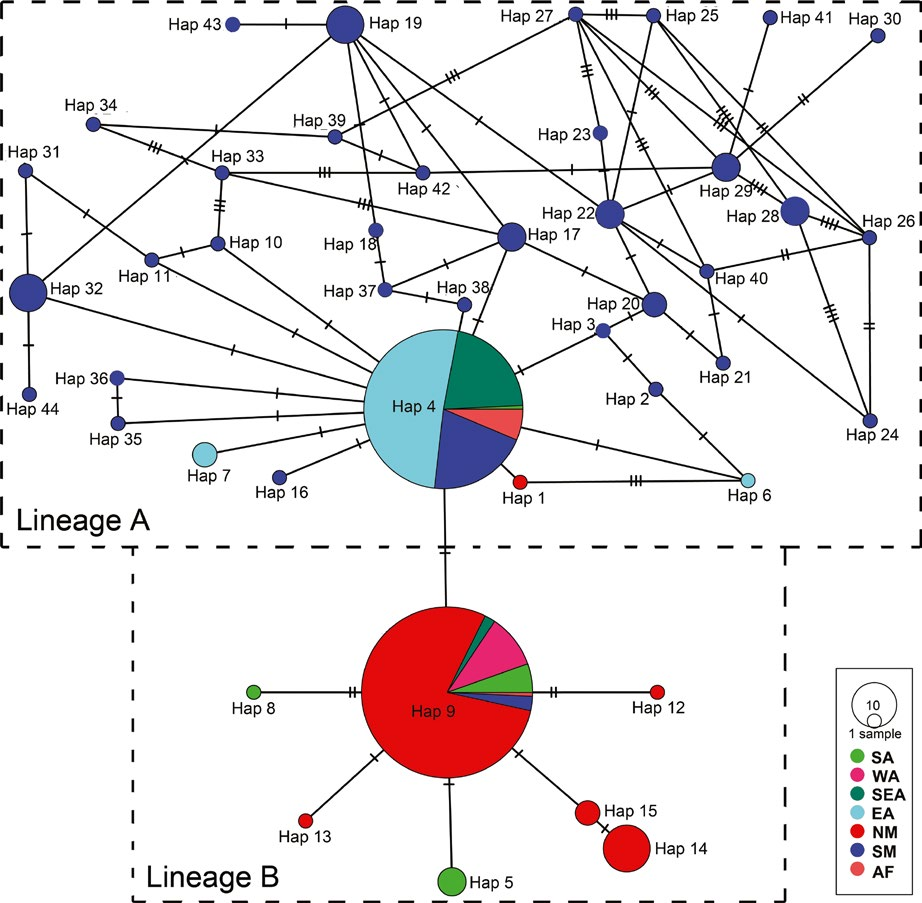
Haplotype network derived from partial sequences of the *COI* gene from the global *Diaphorina citri* populations, built using PopART program. Circle size is proportional to haplotype frequency. SA, South Asia; WA, West Asia; SEA, Southeast Asia; EA, East Asia; NM, North America; SM, South America; AF, Africa

**Figure 3:**
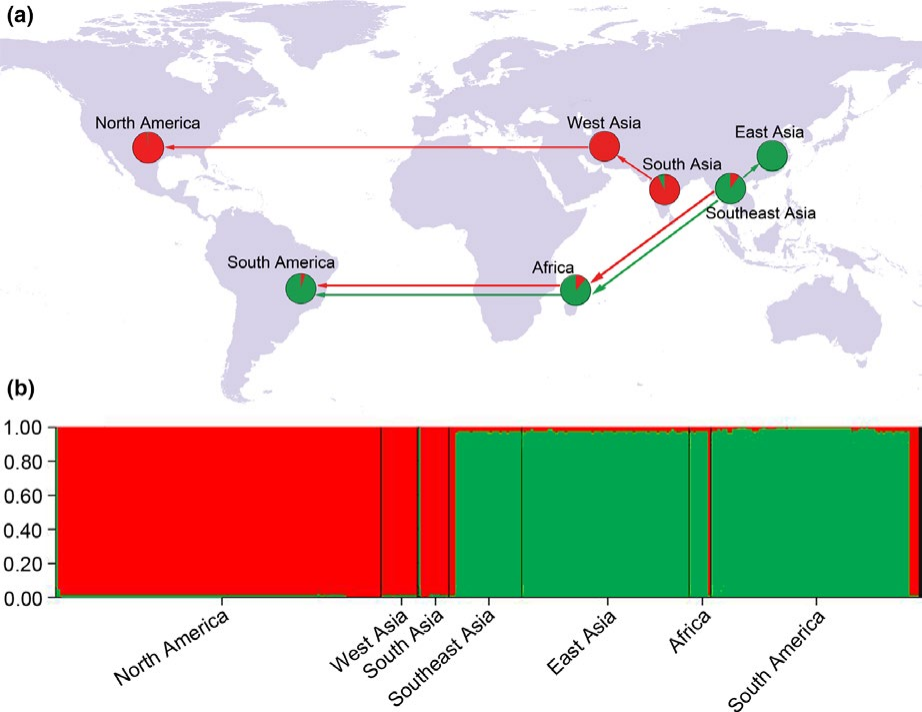
(a) Sampling regions of the Asian citrus psyllid. Detailed sampling information is presented in Table S1 in Appendix S1. A proposed scenario of early dispersal routes is illustrated with arrows. (b) Assignment of 356 individuals to *K* = 2 genetic clusters inferred from STRUCTURE simulations using the mitochondrial *COI* gene sequences. Green represents the lineage A; Red represents the lineage B

We sampled *D. citri* in China between 2015 and 2016. A total of 412 specimens were taken from 11 sampling sites (Table S1 in Appendix S1). Collected insects were stored in 95% alcohol at −20°C. In total, 48 samples representing all our sampling sites were sequenced for genetic analyses. Additional sequences of *D. citri* from Iran, Pakistan, Saudi Arabia, India, Vietnam, Thailand, Indonesia, China, Reunion, Mauritius, Guadeloupe, Puerto Rico, Mexico, USA, and Brazil were taken from GenBank. We analyzed the *COI* sequences of the pest from a total of 356 colonies from 106 geographic sites in Asia, Africa, and America (Figures 2 and 3, and Table S1 in Appendix S1).

The primers (DCITRI *COI*‐L: 5′‐AGGAGGTGGAGACCCAATCT‐3′; DCITRI *COI*‐R: 5′‐TCAATTGGGGGAGAGTTTTG‐3′, Boykin et al., 2012) were used to amplify the *COI* gene from *D. citri*. The 25* *μl polymerase chain reaction (PCR) was run with the following temperature cycling profile: 5 min denaturation at 92°C followed by 35 cycles of 1 min at 92°C denaturation, 1 min at 53°C annealing, 1.5 min at 72°C extension and a final extension at 70°C for 10 min. The PCR reactions were composed of 0.1 mmol/L dNTPs, 2 pmol of each primer, 1.5 U Taq polymerase (Beijing TransGen Biotech Co., Ltd., China), 1× PCR buffer, 2.5 mmol/L MgCl_2_, and 20 ng of DNA. Amplification products were purified and sequenced with primers from the original PCR reactions on an ABI 3730 automatic sequencer (Applied Biosystems, Foster City, CA, USA), using BigDye technology. The chromatographs were interpreted with Phred and Phrap (Green, 2009; Green & Ewing, 2002) using the Chromaseq module (Maddison & Maddison, 2011a) implemented in the Mesquite (Maddison & Maddison, 2011b). Quality threshold was set high, for base trimming to 49 and for calls of ambiguity minimum secondary peak fraction for ambiguity at 0.3. Sequences were checked for errors and edited manually with the program BioEdit (Hall, 1999) and then aligned in both CLUSTALX (Jeanmougin, Thompson, Gouy, Higgins, & Gibson, 1998) under default parameters and MAFFT (http://mafft.cbrc.jp/alignment/server/) using the FFT‐NS‐i strategy to increase accuracy (Katoh, Kuma, Toh, & Miyata, 2005).

### 2.2 Genetic diversity and differentiation

In total, 356 *COI* sequences of the Asian citrus psyllids from Asia, Africa, and America were included in the analyses. Pairwise genetic distances (p‐distances) were calculated using the software MEGA v.5.05 (Tamura et al., 2011). Polymorphic sites, parsimony informative sites, and haplotype frequencies were obtained, and nucleotide (π) and haplotype (H) diversities were estimated as defined by Nei, Muruyama, and Chakraborty (1975) using the DnaSP ver. 5.10 software (Librado & Rozas, 2009). The genetic variability within the whole sample, and among lineages, was assessed by analysis of molecular variance (AMOVA) with the program Arlequin ver. 3.5.1.2 (Excoffier & Lischer, 2010). The estimation of nonsynonymous (Ka) and synonymous (Ks) substitution rates has been used as a way of understanding the evolutionary dynamics of protein‐coding genes across populations (Fay & Wu, 2003; Ohta, 1995). Ks and Ka values were calculated in MEGA V.5 (Tamura et al., 2011) using the Kimura 2P model.

### 2.3 Haplotype network construction and STRUCTURE simulation

A haplotype network was constructed to compare genetic relationships between geographic populations based on the genetic data. The unrooted minimum spanning tree (MST) was implemented in PopART (http://popart.otago.ac.nz) and was partitioned by defined seven populations from the regions: North America (NM); South America (SA); West Asia (WA); East Asia (EA); South Asia (SA); Southeast Asia (SEA); and Africa (AF).

The population substructure among the samples from the analyzed seven regions was inferred using the software STRUCTURE ver. 2.3.3 (Pritchard, Stephens, & Donnelly, 2000). We performed the analysis based on the single‐nucleotide polymorphism (SNP) of *COI* datasets of all individuals sampled (*K* = 1–10) using the admixture model. Ten independent runs were evaluated for each *K*. The total length of the Markov Chain was set to be 100 000, and the length of burn‐in period was 20 000. We adopted the parsimony criterion, in which the lowest *K* capturing the most differentiation among populations was selected as the most likely *K* value (DiLeo, Row, & Lougheed, 2010).

### 2.4 Demographic analysis

To test signatures of demographic changes in *D. citri*, we first examined Tajima's *D* (Posada, 2008) and Fu's *F*_s_ (Clement, Posada, & Crandall, 2000) using Arlequin ver. 3.5.1.2 under the infinite site model with 10,000 coalescent simulations. Second, population demographic changes were also examined by estimating the Harpending's raggedness index (HR) based on mismatch distribution for each population with significance assessed using Arlequin by 1,000 permutations. A significant HR value (*p* < .05) is taken as evidence for rejecting the sudden population expansion model (Schneider & Excoffier, 1999). Third, the demographic history of whether *D. citri* experienced a range expansion was investigated by the spatial expansion model in Arlequin (Schneider & Excoffier, 1999). Mismatch distributions of pairwise differences among sequences were calculated with parametric bootstrapping (1,000 replicates). According to simulations, demographically stable or admixed populations must present a multimodal distribution, whereas populations that have undergone a recent expansion generally show a unimodal distribution (Rogers & Harpending, 1992).

## 3 RESULT

### 3.1 Genetic variability and differentiation

We succeeded in amplifying a 760‐bp *COI* region for the specimens from China and found only one polymorphic site, which was nonsynonymous mutation site. Two unique haplotypes were identified (GenBank Accession number: *submitted*), with relative frequencies of 93.75% and 6.25%, respectively. The *COI* gene comprised 468 bp when including data from GenBank. Forty‐four haplotypes were identified with 31 polymorphic sites and 15 parsimony informative sites. The average p‐distance among *COI* gene sequences was 0.003 (range from 0.000 to 0.017). High haplotype diversity (H‐mean = 0.702 ± 0.017) and low nucleotide diversity (π‐mean = 0.003) were identified within the whole sample. The average number of nucleotide differences (*k*) is 1.483.

Among the 44 unique haplotypes, most (95.45%) occurred only in a single locality, and the remaining 4.55% occurred in more than one locality (Table 1). Hap9 was the most abundant and widely distributed, representing 41.29% of the whole sample, and occurring in all regions expect East Asia. Hap4 was the second most frequent haplotype (35.67%) and was found in four regions but not in West Asia or North America. Hap14 (3.09%) was only found in North America and the remaining haplotypes were restricted to one region (Table 1).

**Table 1:** Region, country of collections, number of individuals, haplotypes (number of individuals), and nucleotide and haplotype diversity of *Diaphorina citri* in each sampled region

Region	Country of collections	Number of individuals	Haplotypes (number of individuals)	Nucleotide diversity (H)	Haplotype diversity ± *SD* (π)
South Asia	India, Pakistan	14	H4(1), H5(4) H8(1), H9(8)	0.00128	0.641 ± 0.097
West Asia	Iran, Saudi Arabia	15	H9(15)	0.00000	0.000 ± 0.000
Southeast Asia	Vietnam, Thailand, Indonesia	30	H4(27), H9(3)	0.00057	0.186 ± 0.088
East Asia	China	69	H4(65), H6(1), H7(3)	0.00040	0.191 ± 0.063
North America	USA, Mexico, Guadeloupe, Puerto Rico	133	H1(1), H9(116), H12(1), H13(1), H14(11), H15(3)	0.00169	0.307 ± 0.050
South America	Brazil	86	H2(1), H3(1), H4(26), H9(4), H10(1), H11(1), H16(1), H17(4), H18(1), H19(7), H20(3), H21(1), H22(4), H23(1), H24(1), H25(1), H26(1), H27(1), H28(1), H29(4), H30(1), H31(1), H32(7), H33(1), H34(1), H35(1), H36(1), H37(1), H38(1), H39(1), H40(1), H41(1), H42(1), H43(1), H44(1)	0.00537	0.892 ± 0.027
Africa	Reunion, Mauritius	9	H4(8), H9(1)	0.00068	0.222 ± 0.166

The overall nonhierarchical AMOVA test (Table 2) resulted in a significant *F*_ST_ value (*F*_ST_ = 0.50, *p* < .0001), indicating the genetic structure among sites. The hierarchical AMOVA (Table 2) analysis between these two lineages resulted in a high *F*_ST_ = 0.62 (*p* < .0001), which demonstrates that global *D. citri* populations are genetically structured. The Ka/Ks ratio is 32.92 (much greater than 1) suggesting natural selection is acting to promote the fixation of advantageous mutations (positive selection).

**Table 2:** Analysis of molecular variance (AMOVA) for *Diaphorina citri* samples using *COI* sequences

Source of variation	*df*	Sum of squares	Variance components	Percentage of variation
The whole sample				
Among populations	6	115.13	0.42	49.72
Within populations	349	148.12	0.42	50.28
			*F*_ST_: 0.50 *p* = .00	
Two lineages				
Among lineages	1	99.18	0.49	47.72
Among populations within lineages	10	30.49	0.14	14.16
Within lineages	344	133.58	0.39	38.12
			*F*_SC_: 0.27 *p* = .00	
			*F*_ST_: 0.62 *p* = .00	
			*F*_CT_: 0.48 *p* = .00	

### 3.2 Global relationships

A haplotype network analysis revealed the genetic structuring among global *D. citri* populations (Figure 2). All individuals of *D. citri* clustered into two matrilines. Lineage A is comprised of 37 haplotypes from all regions expect for West Asia, forming a complex and intricate star‐like connection pattern with Hap4 at the center. Hap4 is widespread in East Asia, Southeast Asia, South Asia, Africa, and South America yet absent from the North American and West Asian samples. Hap1, and Hap6 and Hap7 are from North America and East Asia, respectively. In the A lineage, other haplotypes were restricted to South America. For the lineage B, seven haplotypes were sampled from all regions except East Asia, showing a relatively simple star‐like cluster pattern with Hap9 at the center. Rare haplotypes are one or two mutations from Hap9. Hap9 covered the entire range of lineage B, Hap5 and Hap8 were restricted to South Asia, while all other haplotypes came from North America. The probabilities of each individual belonging to a lineage were plotted by locality (Figure 3a), indicating geographic structuring. The STRUCTURE analysis (Figure 3b) confirmed the presence of two lineages genetically differentiated.

### 3.3 Demographic history

**Figure 4:**
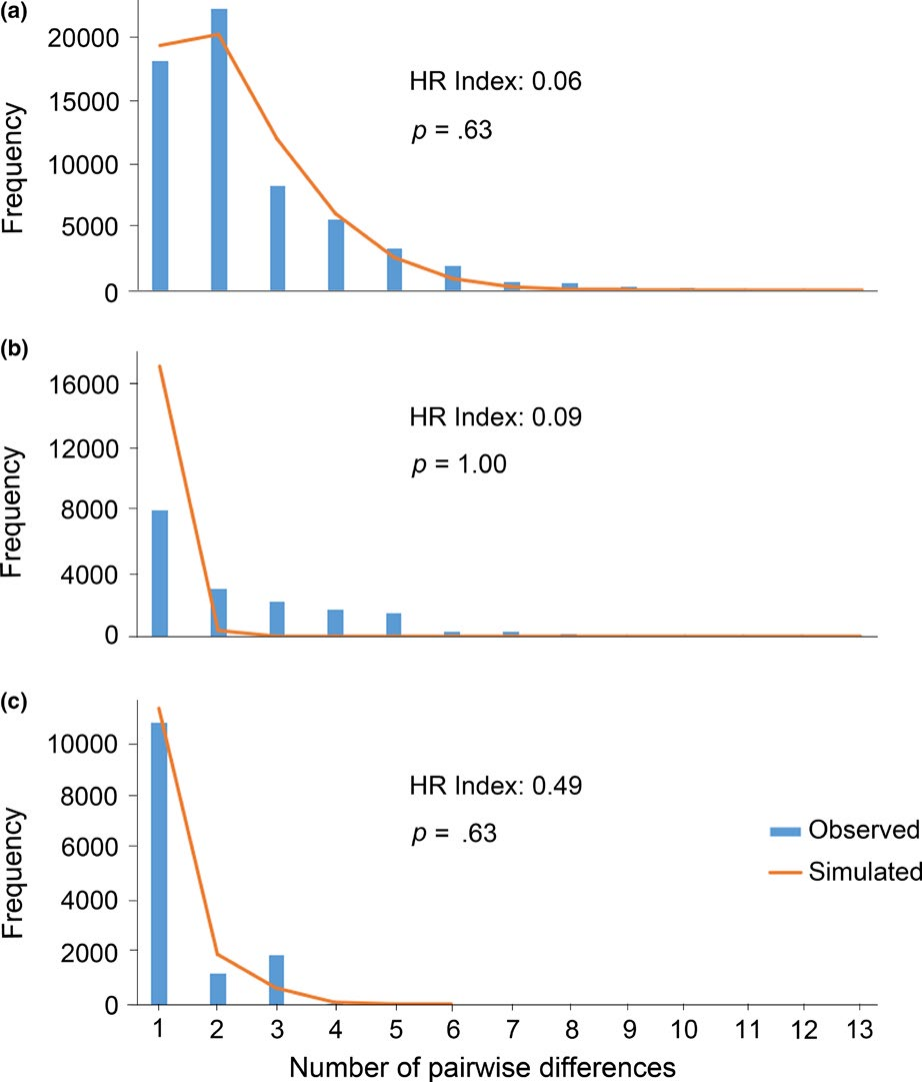
Mismatch distributions of *Diaphorina citri* from the whole sample (a), and from lineage A (b) and B (c) independently

The whole sample and both lineages had significantly negative values of Fu's *F*_s_ (for the whole sample: *F*_s_ = −27.853, *p* = .000; for the lineage A: *F*_s_ = −28.727, *p* = .000; for the lineage B: *F*_s_ = −13.020, *p* = .000) and Tajima's *D* (for the whole sample: *D *=* *−1.865, *p* = .005; for the lineage A: *D *=* *−1.713, *p* = .010; for the lineage B: *D *=* *−1.639, *p* = .017). These statistical values are indicative of historical population expansions, genetic drift, and/or selection (Fu, 1997; Tajima, 1989). Furthermore, haplotype network analyses using the genetic data for *D. citri* further suggested rapid population expansion in that two universal haplotypes located at the central positions of two connected star‐like clusters of rare haplotypes for both lineages (Figure 2). The whole sample and both lineages showed unimodal patterns of mismatch distribution curves and the strong biases toward low divergence values as distinguished by 0‐ and 1‐nucleotide changes (Figure 4). Furthermore, none of the Harpending's raggedness (HR) index of mismatch distribution was significant (Figure 4). These findings illustrated a relatively recent and rapid population expansion from a small number of colonizers (Rogers & Harpending, 1992) or a range expansion with high levels of migration between neighboring demes (Excoffier, 2004; Ray, Currat, & Excoffier, 2003).

## 4 DISCUSSION

### 4.1 Genetic diversity and differentiation

We present the most inclusive analysis of the mtDNA phylogeographic structure of *D. citri* to date, collating previously published *COI* data (Boykin et al., 2012; de León et al., 2011; Guidolin, Fresia, & Cônsoli, 2014; Lashkari et al., 2014) with novel samples from Asia. Our phylogenetic network analysis inferred by the *COI* dataset suggests two lineages of *D. citri* in the world, here dubbed A and B. The genetic diversity of lineage A was higher than that of lineage B. Moreover, different genetic diversity (H and π) levels for *D. citri* were seen in the seven analyzed regions. The psyllids from both East and West Asia, and North America showed low genetic diversity, results that could imply recent introductions events that may have consisted of as little as a single haplotype. We cannot rule out other processes such as reduction in mtDNA variation due to *Wolbachia* infections, male‐biased dispersal, or other potential limitations of mtDNA data (Avise, 2000). However, our findings seem more likely to reflect colonization histories and are consistent with genetic diversity of introduced populations being low due to the small size of propagules and bottleneck effects (Nei et al., 1975). South America, we hypothesize, was colonized recently by *D*. *citri*, yet it displays high mtDNA diversity. This could be the result of multiple invasions and/or a relative longer expansion history, consistent with prior studies (Guidolin et al., 2014; Silva et al., 1968). The genetic diversity of *D. citri* has been studied in Iran and Pakistan (Lashkari et al., 2014), Brazil (Guidolin, Fresia, & Cônsoli, 2014), America (de León et al., 2011) and worldwide (Boykin et al., 2012) using the same *COI* region we have applied. However, the current study extends on prior studies by combining all existing *COI* data and further adds novel samples from various localities in China. Consequently, our analyses reveal a more comprehensive insight into global mtDNA diversity than prior studies. Boykin et al. (2012) identified only eight haplotypes from worldwide *D. citri* populations, compared to the 44 discussed here. Many of the haplotypes of *D. citri* we find abundant were not present in the study of Boykin et al. (2012), meaning that with the current analysis we have gained a deeper insight into the global genetic variation and distribution of the psyllid.

The results of the AMOVA analysis demonstrated significant geographic structuring of genetic variation among populations within as well as between lineages. We also found strong genetic differentiation between the two lineages even though they do co‐occur in some localities. These results indicate limited or no mtDNA gene flow between the lineages, and limited gene flow within lineages among geographically isolated populations. On this basis, we hypothesize that this species has limited natural (nonhuman mediated) dispersal capacity (at least females, Kobori, Nakata, Ohto, & Takasu, 2011), and therefore, local populations may be unique and amenable to site‐specific containment strategies to reduce the citrus greening disease spread. This observed geographic structure of *D. citri* is consistent with a process of range expansion followed by isolation of populations, as demonstrated in Excoffier, Foll, and Petit (2009) studies.

The observed significant positive selection (Ka/Ks ≫ 1) here is interesting as invasion success may occur after severe population bottleneck events or even with singly mated female invasion depending on its heterozygosity level (Zayed, Constantin, & Parker, 2007). In recent years, massive pesticides were used to control *D. citri* in the citrus orchards (Lopes, Frare, Yamamoto, Ayres, & Barbosa, 2007; Yamamoto et al., 2009). Therefore, the pest has been undergoing the intense selective pressure. Additionally, rapidly changing environments in invasive regions, such as climate change, also produce strong selective pressure. These directional selection can quickly lead to the loss of haplotypes and the expansion of rare haplotypes with different fitness attributes, affecting management strategies to be adopted for controlling the pest *D. citri* and/or the citrus greening disease.

### 4.2 Dispersal history

Our mtDNA phylogeographic analysis suggests two haplotype lineages, each characterized by a common “ancestral” haplotype and several derivatives of it (Figure 2). Although several potential processes might yield such a pattern (as outlined above), we hypothesize based on these results that the two lineages have independent colonization histories from two separate ancestral haplotypes. We hypothesize three dispersal routes across the globe, in part showing parallel dispersal patterns for the two lineages in Southeast Asia, Africa, and South America. The genealogies based on network can provide insight into ancestral haplotypes, as these haplotypes are expected to be common, widely distributed and highly connected (Chen et al., 2010). In the study by Boykin et al. (2012), >60% of the analyzed individuals were sampled from the New World where *D. citri* is invasive. These sampling limitations hinder reconstruction of ancestral haplotypes and potential dispersal routes. Our analysis offers a broader and more balanced sampling of *D. citri* allowing a better prediction of ancestry and dispersal. The haplotype network analysis identified two ancestral haplotypes (Hap4, lineage A; Hap9, lineage B, Figure 2) that although genetically close are the unequivocal connection points of all other haplotypes, supporting our hypothesis of independent histories.

Mead (1977) listed the Far East as the geographic origin of the Asian citrus psyllid. More recent studies on biogeographic reconstructions inferred from origins of plant host and historical information indicate that the geographic origin of this psyllid may be southern Asia, probably India (Hall, 2008). Our findings are consistent with the latter; a southern and/or southeastern Asia origin. The Asian citrus psyllid is known to occur in China likely as an invasion within the last 80 years (Hoffmann, 1936). It colonized South America in the 1940s (Costa Lima, 1942). During the 1990s, *D. citri* invaded North America (French, Kahlke, & De Graca, 2001; Halbert & Núñez, 2004; Pluke, Qureshi, & Stansly, 2008; Tsai, Wang, & Liu, 2000).

The introductions and dispersal patterns of *D. citri* in the Americas were recently studied using *COI* data. de León et al. (2011) suggested that two separate invasions or founding events of the Asian citrus psyllid occurred respectively in South America and North America. Guidolin, Fresia and Cônsoli (2014) argued for two hypothetical scenarios of invasions for *D. citri* in Brazil. The first scenario is that the psyllid invaded from the eastern region of the state to São Paulo, followed by range expansion to the centralwestern regions. The second is that invasion of bordering states into the western region of São Paulo and then expanded to the central and eastern regions. But to date, no clear hypotheses exist regarding the global invasion history of *D. citri*. Based on results of this study, the haplotype frequency, the phylogeographical distribution, and the patterns of haplotype connectivity, we hypothesize three colonization scenarios: the expansion of lineage B from southern Asia into North America via West Asia, the expansion of lineage A from Southeast Asia into East Asia, and the parallel dispersals of the two lineages into Africa and South America (Figure 3a). In addition, the parallel dispersal patterns may involve multiple propagules, which might explain the high genetic diversity observed in South America, and the finding of an individual of lineage B in northern America where lineage A dominates.

Significantly negative neutrality tests (Tajima's *D* and Fu's *F*_s_) within each lineage of *D. citri*, two star‐like cluster patterns in the haplotype network, and the unimodal mismatch distributions for the whole sample and both lineages all rejected the null hypothesis of neutral evolution of the partial *COI* gene. That low genetic diversity characterized most sampling regions, and native and invasive populations share common haplotypes (Hap4 and Hap9), is consistent with recent introductions events and could suggest a single introduction of haplotypes in most region.

### 4.3 Future directions

The continent‐global scale phylogeographic history of the psyllid *D. citri* has to date exclusively been studied with mtDNA represented by the *COI* locus. These studies have allowed understanding of the matrilineage structure of this species and generated hypotheses regarding dispersal and colonization of this important pest. However, while mtDNA data have proven highly useful in phylogeographic studies, there are several shortcomings of this approach (e.g., Avise, 2000). Not the least of these is the potential mismatch between the dispersal history of males and females and likely mismatch between a species tree and any single gene tree. With this paper, we summarize available *COI* data and add new data re‐analyses of global patterns, and discuss prior and novel hypotheses on dispersal and colonization history of *D. citri*. Robustly testing these hypotheses, and gaining a deeper understanding of population structure of this species, will require data drawn at random from the whole genome, utilizing methods such as RADseq or targeted sequence capture. Such data will tease apart male and female dispersal, testing the “female‐based” dispersal hypotheses proposed here. They will also allow testing of “independent lineages” hypothesis through precise measures of genetic isolation and gene flow unavailable to single‐marker studies. Finally, such data will be immune to potential issues such as *Wolbachia* induced biases. However, for such work to be realized, resampling specimens across the globe will be necessary, and we hope our study can aid in strategical sampling reflecting known haplotype diversity.

## CONFLICT OF INTEREST

None declared.

## AUTHOR CONTRIBUTIONS

Yufa Luo designed this study, collected and processed samples from all field sites, conducted data analyses, and wrote the manuscript. Ingi Agnarsson helped extensively with manuscript writing. All authors critically revised and approved the final version of the manuscript submitted.
