# Supplementary material for: Global mtDNA genetic structure and hypothesized invasion history of a major pest of citrus, Diaphorina citri (Hemiptera: Liviidae)
Source: Ecol Evol. 2017 Nov 26;8(1):257–65. doi: 10.1002/ece3.3680 (PMC5756889; doi:10.1002/ece3.3680)
Supplement: Supplementary file 1 [file ECE3-8-257-s001.doc]

**Supplementary material Appendix 1**

**TABLE 1** Locations and accession numbers of *Diaphorina citri*

| Number | GenBank code | Country | Locality | *COI* |
| --- | --- | --- | --- | --- |
| 1 | psy57-5 | Vietnam | My Tho | FJ190382 |
| 2 | psy57-4 | Vietnam | My Tho | FJ190381 |
| 3 | psy57-3 | Vietnam | My Tho | FJ190380 |
| 4 | psy57-2 | Vietnam | My Tho | FJ190379 |
| 5 | psy57-1 | Vietnam | My Tho | FJ190378 |
| 6 | psy56-5 | USA | Immokalee, Florida | FJ190377 |
| 7 | psy56-3 | USA | Immokalee, Florida | FJ190376 |
| 8 | psy56-2 | USA | Immokalee, Florida | FJ190375 |
| 9 | psy56-1 | USA | Immokalee, Florida | FJ190374 |
| 10 | psy55-5 | USA | Immokalee, Florida | FJ190373 |
| 11 | psy55-4 | USA | Immokalee, Florida | FJ190372 |
| 12 | psy55-2 | USA | Immokalee, Florida | FJ190371 |
| 13 | psy55-1 | USA | Immokalee, Florida | FJ190370 |
| 14 | psy54-5 | China | Guangzhou | FJ190369 |
| 15 | psy54-4 | China | Guangzhou | FJ190368 |
| 16 | psy54-2 | China | Guangzhou | FJ190367 |
| 17 | psy54-1 | China | Guangzhou | FJ190366 |
| 18 | psy52-4 | China | Fuzhou | FJ190365 |
| 19 | psy52-3 | China | Fuzhou | FJ190364 |
| 20 | psy52-2 | China | Fuzhou | FJ190363 |
| 21 | psy52-1 | China | Fuzhou | FJ190362 |
| 22 | psy51-5 | China | Fuzhou | FJ190361 |
| 23 | psy51-4 | China | Fuzhou | FJ190360 |
| 24 | psy51-3 | China | Fuzhou | FJ190359 |
| 25 | psy51-2 | China | Fuzhou | FJ190358 |
| 26 | psy51-1 | China | Fuzhou | FJ190357 |
| 27 | psy50-5 | Guadeloupe | - | FJ190356 |
| 28 | psy50-2 | Guadeloupe | - | FJ190355 |
| 29 | psy50-1 | Guadeloupe | - | FJ190354 |
| 30 | psy49-5 | Guadeloupe | - | FJ190353 |
| 31 | psy49-4 | Guadeloupe | - | FJ190352 |
| 32 | psy49-3 | Guadeloupe | - | FJ190351 |
| 33 | psy49-1 | Guadeloupe | - | FJ190350 |
| 34 | psy48-4 | Guadeloupe | - | FJ190349 |
| 35 | psy48-3 | Guadeloupe | - | FJ190348 |
| 36 | psy48-2 | Guadeloupe | - | FJ190347 |
| 37 | psy48-1 | Guadeloupe | - | FJ190346 |
| 38 | psy47-5 | India | - | FJ190345 |
| 39 | psy47-4 | India | - | FJ190344 |
| 40 | psy47-2 | India | - | FJ190343 |
| 41 | psy47-1 | India | - | FJ190342 |
| 42 | psy46-5 | Saudi Arabia | - | FJ190341 |
| 43 | psy46-4 | Saudi Arabia | - | FJ190340 |
| 44 | psy46-3 | Saudi Arabia | - | FJ190339 |
| 45 | psy46-2 | Saudi Arabia | - | FJ190338 |
| 46 | psy46-1 | Saudi Arabia | - | FJ190337 |
| 47 | psy45-3 | Indonesia | Bali | FJ190336 |
| 48 | psy45-2 | Indonesia | Bali | FJ190335 |
| 49 | psy45-1 | Indonesia | Bali | FJ190334 |
| 50 | psy44-5 | Brazil | - | FJ190333 |
| 51 | psy44-4 | Brazil | - | FJ190332 |
| 52 | psy44-3 | Brazil | - | FJ190331 |
| 53 | psy44-2 | Brazil | - | FJ190330 |
| 54 | psy44-1 | Brazil | - | FJ190329 |
| 55 | psy43-5 | Brazil | - | FJ190328 |
| 56 | psy43-3 | Brazil | - | FJ190327 |
| 57 | psy43-2 | Brazil | - | FJ190326 |
| 58 | psy43-1 | Brazil | - | FJ190325 |
| 59 | psy42-5 | Brazil | Bresil | FJ190324 |
| 60 | psy42-4 | Brazil | Bresil | FJ190323 |
| 61 | psy42-3 | Brazil | Bresil | FJ190322 |
| 62 | psy42-2 | Brazil | Bresil | FJ190321 |
| 63 | psy41-4 | Reunion | - | FJ190320 |
| 64 | psy41-3 | Reunion | - | FJ190319 |
| 65 | psy41-2 | Reunion | - | FJ190318 |
| 66 | psy41-1 | Reunion | - | FJ190317 |
| 67 | psy40-5 | Mauritius | - | FJ190316 |
| 68 | psy40-4 | Mauritius | - | FJ190315 |
| 69 | psy40-3 | Mauritius | - | FJ190314 |
| 70 | psy40-2 | Mauritius | - | FJ190313 |
| 71 | psy40-1 | Mauritius | - | FJ190312 |
| 72 | psy39-5 | USA | Florida | FJ190311 |
| 73 | psy39-2 | USA | Florida | FJ190310 |
| 74 | psy38-5 | Mexico | Nuevo Leon | FJ190309 |
| 75 | psy38-4 | Mexico | Nuevo Leon | FJ190308 |
| 76 | psy38-3 | Mexico | Nuevo Leon | FJ190307 |
| 77 | psy38-1 | Mexico | Nuevo Leon | FJ190306 |
| 78 | psy37-3 | Mexico | Yucatan | FJ190305 |
| 79 | psy37-2 | Mexico | Yucatan | FJ190304 |
| 80 | psy37-1 | Mexico | Yucatan | FJ190303 |
| 81 | psy36-3 | Mexico | Yucatan | FJ190302 |
| 82 | psy36-2 | Mexico | Yucatan | FJ190301 |
| 83 | psy36-1 | Mexico | Yucatan | FJ190300 |
| 84 | psy35-5 | China | Zhejiang | FJ190299 |
| 85 | psy35-4 | China | Zhejiang | FJ190298 |
| 86 | psy35-2 | China | Zhejiang | FJ190297 |
| 87 | psy34-5 | Thailand | Hat Yai | FJ190296 |
| 88 | psy34-4 | Thailand | Hat Yai | FJ190295 |
| 89 | psy34-3 | Thailand | Hat Yai | FJ190294 |
| 90 | psy34-2 | Thailand | Hat Yai | FJ190293 |
| 91 | psy33-5 | Pakistan | - | FJ190292 |
| 92 | psy33-4 | Pakistan | - | FJ190291 |
| 93 | psy33-3 | Pakistan | - | FJ190290 |
| 94 | psy33-2 | Pakistan | - | FJ190289 |
| 95 | psy33-1 | Pakistan | - | FJ190288 |
| 96 | psy32-5 | China | Taipei, Taiwan | FJ190287 |
| 97 | psy32-4 | China | Taipei, Taiwan | FJ190286 |
| 98 | psy32-3 | China | Taipei, Taiwan | FJ190285 |
| 99 | psy32-2 | China | Taipei, Taiwan | FJ190284 |
| 100 | psy32-1 | China | Taipei, Taiwan | FJ190283 |
| 101 | psy31-5 | Indonesia | Candisari | FJ190282 |
| 102 | psy31-3 | Indonesia | Candisari | FJ190281 |
| 103 | psy31-2 | Indonesia | Candisari | FJ190280 |
| 104 | psy31-1 | Indonesia | Candisari | FJ190279 |
| 105 | psy30-3 | USA | Florida | FJ190278 |
| 106 | psy30-1 | USA | Florida | FJ190277 |
| 107 | psy29-5 | Vietnam | Hanoi | FJ190276 |
| 108 | psy29-4 | Vietnam | Hanoi | FJ190275 |
| 109 | psy29-3 | Vietnam | Hanoi | FJ190274 |
| 110 | psy29-2 | Vietnam | Hanoi | FJ190273 |
| 111 | psy29-1 | Vietnam | Hanoi | FJ190272 |
| 112 | psy28-5 | Indonesia | Banjer Bantes, Bali | FJ190271 |
| 113 | psy28-4 | Indonesia | Banjer Bantes, Bali | FJ190270 |
| 114 | psy28-3 | Indonesia | Banjer Bantes, Bali | FJ190269 |
| 115 | psy28-2 | Indonesia | Banjer Bantes, Bali | FJ190268 |
| 116 | psy28-1 | Indonesia | Banjer Bantes, Bali | FJ190267 |
| 117 | psy27-2 | Indonesia | Banjer Bantes, Bali | FJ190266 |
| 118 | psy26-5 | Indonesia | Java | FJ190265 |
| 119 | psy26-4 | Indonesia | Java | FJ190264 |
| 120 | psy26-2 | Indonesia | Java | FJ190263 |
| 121 | psy24-5 | Puerto Rico | Corazal | FJ190262 |
| 122 | psy24-4 | Puerto Rico | Corazal | FJ190261 |
| 123 | psy24-3 | Puerto Rico | Corazal | FJ190260 |
| 124 | psy23-5 | USA | Florida | FJ190259 |
| 125 | psy23-4 | USA | Florida | FJ190258 |
| 126 | psy23-3 | USA | Florida | FJ190257 |
| 127 | psy23-1 | USA | Florida | FJ190256 |
| 128 | psy22-5 | USA | Florida | FJ190255 |
| 129 | psy22-4 | USA | Florida | FJ190254 |
| 130 | psy22-3 | USA | Florida | FJ190253 |
| 131 | psy22-2 | USA | Florida | FJ190252 |
| 132 | psy22-1 | USA | Florida | FJ190251 |
| 133 | psy21-5 | USA | Florida | FJ190250 |
| 134 | psy21-4 | USA | Florida | FJ190249 |
| 135 | psy21-3 | USA | Florida | FJ190248 |
| 136 | psy21-2 | USA | Florida | FJ190247 |
| 137 | psy21-1 | USA | Florida | FJ190246 |
| 138 | psy20-5 | USA | Florida | FJ190245 |
| 139 | psy20-4 | USA | Florida | FJ190244 |
| 140 | psy20-2 | USA | Florida | FJ190243 |
| 141 | psy20-1 | USA | Florida | FJ190242 |
| 142 | psy19-5 | USA | Florida | FJ190241 |
| 143 | psy19-4 | USA | Florida | FJ190240 |
| 144 | psy19-3 | USA | Florida | FJ190239 |
| 145 | psy19-2 | USA | Florida | FJ190238 |
| 146 | psy19-1 | USA | Florida | FJ190237 |
| 147 | psy18-5 | USA | Florida | FJ190236 |
| 148 | psy18-4 | USA | Florida | FJ190235 |
| 149 | psy18-3 | USA | Florida | FJ190234 |
| 150 | psy18-2 | USA | Florida | FJ190233 |
| 151 | psy18-1 | USA | Florida | FJ190232 |
| 152 | psy17-4 | Brazil | Piraciba | FJ190231 |
| 153 | psy17-3 | Brazil | Piraciba | FJ190230 |
| 154 | psy17-2 | Brazil | Piraciba | FJ190229 |
| 155 | psy17-1 | Brazil | Piraciba | FJ190228 |
| 156 | psy16-5 | USA | Florida | FJ190227 |
| 157 | psy16-1 | USA | Florida | FJ190226 |
| 158 | psy15-5 | USA | Florida | FJ190225 |
| 159 | psy15-4 | USA | Florida | FJ190224 |
| 160 | psy15-3 | USA | Florida | FJ190223 |
| 161 | psy15-2 | USA | Florida | FJ190222 |
| 162 | psy15-1 | USA | Florida | FJ190221 |
| 163 | psy14-5 | USA | Florida | FJ190220 |
| 164 | psy14-4 | USA | Florida | FJ190219 |
| 165 | psy14-3 | USA | Florida | FJ190218 |
| 166 | psy14-2 | USA | Florida | FJ190217 |
| 167 | psy14-1 | USA | Florida | FJ190216 |
| 168 | psy13-5 | USA | Florida | FJ190215 |
| 169 | psy13-4 | USA | Florida | FJ190214 |
| 170 | psy13-3 | USA | Florida | FJ190213 |
| 171 | psy13-2 | USA | Florida | FJ190212 |
| 172 | psy13-1 | USA | Florida | FJ190211 |
| 173 | psy12-5 | USA | Florida | FJ190210 |
| 174 | psy12-4 | USA | Florida | FJ190209 |
| 175 | psy12-2 | USA | Florida | FJ190208 |
| 176 | psy12-1 | USA | Florida | FJ190207 |
| 177 | psy11-5 | USA | Florida | FJ190206 |
| 178 | psy11-4 | USA | Florida | FJ190205 |
| 179 | psy11-3 | USA | Florida | FJ190204 |
| 180 | psy11-2 | USA | Florida | FJ190203 |
| 181 | psy11-1 | USA | Florida | FJ190202 |
| 182 | psy10-5 | USA | Florida | FJ190201 |
| 183 | psy10-4 | USA | Florida | FJ190200 |
| 184 | psy10-3 | USA | Florida | FJ190199 |
| 185 | psy10-2 | USA | Florida | FJ190198 |
| 186 | psy10-1 | USA | Florida | FJ190197 |
| 187 | psy9-5 | USA | Florida | FJ190196 |
| 188 | psy9-4 | USA | Florida | FJ190195 |
| 189 | psy9-3 | USA | Florida | FJ190194 |
| 190 | psy9-2 | USA | Florida | FJ190193 |
| 191 | psy9-1 | USA | Florida | FJ190192 |
| 192 | psy8-5 | USA | Florida | FJ190191 |
| 193 | psy8-4 | USA | Florida | FJ190190 |
| 194 | psy8-3 | USA | Florida | FJ190189 |
| 195 | psy8-2 | USA | Florida | FJ190188 |
| 196 | psy8-1 | USA | Florida | FJ190187 |
| 197 | psy4-5 | USA | Florida | FJ190186 |
| 198 | psy4-4 | USA | Florida | FJ190185 |
| 199 | psy4-3 | USA | Florida | FJ190184 |
| 200 | psy4-2 | USA | Florida | FJ190183 |
| 201 | psy4-1 | USA | Florida | FJ190182 |
| 202 | psy3-5 | USA | Texas | FJ190181 |
| 203 | psy3-4 | USA | Texas | FJ190180 |
| 204 | psy3-3 | USA | Texas | FJ190179 |
| 205 | psy3-2 | USA | Texas | FJ190178 |
| 206 | psy3-1 | USA | Texas | FJ190177 |
| 207 | psy2-5 | USA | Florida | FJ190176 |
| 208 | psy2-4 | USA | Florida | FJ190175 |
| 209 | psy2-3 | USA | Florida | FJ190174 |
| 210 | psy2-2 | USA | Florida | FJ190173 |
| 211 | psy2-1 | USA | Florida | FJ190172 |
| 212 | psy1-5 | USA | St. Lucie County | FJ190171 |
| 213 | psy1-4 | USA | St. Lucie County | FJ190170 |
| 214 | psy1-3 | USA | St. Lucie County | FJ190169 |
| 215 | psy1-2 | USA | St. Lucie County | FJ190168 |
| 216 | psy1-1 | USA | St. Lucie County | FJ190167 |
| 217 | CP30CAMU-58 | Mexico | Cazones, Veracruz | KJ453897 |
| 218 | CP30CANM-74 | Mexico | Cazones, Veracruz | KJ453896 |
| 219 | CP30CAS8-137 | Mexico | Cazones, Veracruz | KJ453895 |
| 220 | CP30CALI-83 | Mexico | Cazones, Veracruz | KJ453894 |
| 221 | CP30CALI-77 | Mexico | Cazones, Veracruz | KJ453893 |
| 222 | CP30CANV-76 | Mexico | Cazones, Veracruz | KJ453892 |
| 223 | CP30CAMU-42 | Mexico | Cazones, Veracruz | KJ453891 |
| 224 | CP30CANV-20 | Mexico | Cazones, Veracruz | KJ453890 |
| 225 | CP30CANM-15 | Mexico | Cazones, Veracruz | KJ453889 |
| 226 | haplotype 47 | Brazil | - | KC354785 |
| 227 | haplotype 46 | Brazil | - | KC354784 |
| 228 | haplotype 45 | Brazil | - | KC354783 |
| 229 | haplotype 44 | Brazil | - | KC354782 |
| 230 | haplotype 43 | Brazil | - | KC354781 |
| 231 | haplotype 42 | Brazil | - | KC354780 |
| 232 | haplotype 41 | Brazil | - | KC354779 |
| 233 | haplotype 40 | Brazil | - | KC354778 |
| 234 | haplotype 39 | Brazil | - | KC354777 |
| 235 | haplotype 38 | Brazil | - | KC354776 |
| 236 | haplotype 37 | Brazil | - | KC354775 |
| 237 | haplotype 36 | Brazil | - | KC354774 |
| 238 | haplotype 35 | Brazil | - | KC354773 |
| 239 | haplotype 34 | Brazil | - | KC354772 |
| 240 | haplotype 33 | Brazil | - | KC354771 |
| 241 | haplotype 32 | Brazil | - | KC354770 |
| 242 | haplotype 31 | Brazil | - | KC354769 |
| 243 | haplotype 30 | Brazil | - | KC354768 |
| 244 | haplotype 29 | Brazil | - | KC354767 |
| 245 | haplotype 28 | Brazil | - | KC354766 |
| 246 | haplotype 27 | Brazil | - | KC354765 |
| 247 | haplotype 26 | Brazil | - | KC354764 |
| 248 | haplotype 25 | Brazil | - | KC354763 |
| 249 | haplotype 24 | Brazil | - | KC354762 |
| 250 | haplotype 23 | Brazil | - | KC354761 |
| 251 | haplotype 22 | Brazil | - | KC354760 |
| 252 | haplotype 21 | Brazil | - | KC354759 |
| 253 | haplotype 20 | Brazil | - | KC354758 |
| 254 | haplotype 19 | Brazil | - | KC354757 |
| 255 | haplotype 18 | Brazil | - | KC354756 |
| 256 | haplotype 17 | Brazil | - | KC354755 |
| 257 | haplotype 16 | Brazil | - | KC354754 |
| 258 | haplotype 15 | Brazil | - | KC354753 |
| 259 | haplotype 14 | Brazil | - | KC354752 |
| 260 | haplotype 13 | Brazil | - | KC354751 |
| 261 | haplotype 12 | Brazil | - | KC354750 |
| 262 | haplotype 11 | Brazil | - | KC354749 |
| 263 | haplotype 10 | Brazil | - | KC354748 |
| 264 | haplotype 9 | Brazil | - | KC354747 |
| 265 | haplotype 8 | Brazil | - | KC354746 |
| 266 | haplotype 7 | Brazil | - | KC354745 |
| 267 | haplotype 6 | Brazil | - | KC354744 |
| 268 | haplotype 5 | Brazil | - | KC354743 |
| 269 | haplotype 4 | Brazil | - | KC354742 |
| 270 | haplotype 3 | Brazil | - | KC354741 |
| 271 | haplotype 2 | Brazil | - | KC354740 |
| 272 | haplotype 1 | Brazil | - | KC354739 |
| 273 | FD2 | Iran | - | KC509572 |
| 274 | FD1 | Iran | - | KC509571 |
| 275 | SB2 | Iran | - | KC509570 |
| 276 | SB1 | Iran | - | KC509569 |
| 277 | HF2 | Iran | - | KC509568 |
| 278 | HF1 | Iran | - | KC509567 |
| 279 | HR2 | Iran | - | KC509566 |
| 280 | HR1 | Iran | - | KC509565 |
| 281 | KJ2 | Iran | - | KC509564 |
| 282 | KJ1 | Iran | - | KC509563 |
| 283 | P2 | Pakistan | - | KC509562 |
| 284 | P1 | Pakistan | - | KC509561 |
| 285 | haplotype 22 | Brazil | - | KC011243 |
| 286 | haplotype 21 | Brazil | - | KC011242 |
| 287 | haplotype 20 | Brazil | - | KC011241 |
| 288 | haplotype 19 | Brazil | - | KC011240 |
| 289 | haplotype 18 | Brazil | - | KC011239 |
| 290 | haplotype 17 | Brazil | - | KC011238 |
| 291 | haplotype 16 | Brazil | - | KC011237 |
| 292 | haplotype 15 | Brazil | - | KC011236 |
| 293 | haplotype 14 | Brazil | - | KC011235 |
| 294 | haplotype 13 | Brazil | - | KC011234 |
| 295 | haplotype 12 | Brazil | - | KC011233 |
| 296 | haplotype 11 | Brazil | - | KC011232 |
| 297 | haplotype 10 | Brazil | - | KC011231 |
| 298 | haplotype 9 | Brazil | - | KC011230 |
| 299 | haplotype 8 | Brazil | - | KC011229 |
| 300 | haplotype 7 | Brazil | - | KC011228 |
| 301 | haplotype 6 | Brazil | - | KC011227 |
| 302 | haplotype 5 | Brazil | - | KC011226 |
| 303 | haplotype 4 | Brazil | - | KC011225 |
| 304 | haplotype 3 | Brazil | - | KC011224 |
| 305 | haplotype 2 | Brazil | - | KC011223 |
| 306 | haplotype 1 | Brazil | - | KC011222 |
| 307 | Andhra Pradesh | India | Mehboob Nagar | KR865960 |
| 308 | Paithan | India | Paithan, Maharashtra | KR865959 |
| 309 | X001 | China | Ruijing, Jiangxi | *submitting* |
| 310 | X002 | China | Ruijing, Jiangxi | *submitting* |
| 311 | X003 | China | Ruijing, Jiangxi | *submitting* |
| 312 | X004 | China | Longnan, Jiangxi | *submitting* |
| 313 | X005 | China | Longnan, Jiangxi | *submitting* |
| 314 | X006 | China | Longnan, Jiangxi | *submitting* |
| 315 | X007 | China | Shicheng, Jiangxi | *submitting* |
| 316 | X008 | China | Shicheng, Jiangxi | *submitting* |
| 317 | X009 | China | Shicheng, Jiangxi | *submitting* |
| 318 | X010 | China | Xingguo, Jiangxi | *submitting* |
| 319 | X011 | China | Xingguo, Jiangxi | *submitting* |
| 320 | X012 | China | Xingguo, Jiangxi | *submitting* |
| 321 | X013 | China | Xingguo, Jiangxi | *submitting* |
| 322 | X014 | China | Chongyi A, Jiangxi | *submitting* |
| 323 | X015 | China | Chongyi B, Jiangxi | *submitting* |
| 324 | X016 | China | Chongyi A, Jiangxi | *submitting* |
| 325 | X017 | China | Chongyi A, Jiangxi | *submitting* |
| 326 | X018 | China | Chongyi B, Jiangxi | *submitting* |
| 327 | X019 | China | Xunwu, Jiangxi | *submitting* |
| 328 | X020 | China | Xunwu, Jiangxi | *submitting* |
| 329 | X021 | China | Xunwu, Jiangxi | *submitting* |
| 330 | X022 | China | Xunwu, Jiangxi | *submitting* |
| 331 | X023 | China | Xunwu, Jiangxi | *submitting* |
| 332 | X024 | China | Xunwu, Jiangxi | *submitting* |
| 333 | X025 | China | Dayu A, Jiangxi | *submitting* |
| 334 | X026 | China | Dayu B, Jiangxi | *submitting* |
| 335 | X027 | China | Dayu A, Jiangxi | *submitting* |
| 336 | X028 | China | Dayu A, Jiangxi | *submitting* |
| 337 | X029 | China | Dayu B, Jiangxi | *submitting* |
| 338 | X030 | China | Dayu A, Jiangxi | *submitting* |
| 339 | X031 | China | Dayu A, Jiangxi | *submitting* |
| 340 | X032 | China | Dayu B, Jiangxi | *submitting* |
| 341 | X033 | China | Zhanggong, Jiangxi | *submitting* |
| 342 | X034 | China | Zhanggong, Jiangxi | *submitting* |
| 343 | X035 | China | Zhanggong, Jiangxi | *submitting* |
| 344 | X036 | China | Zhanggong, Jiangxi | *submitting* |
| 345 | X037 | China | Zhanggong, Jiangxi | *submitting* |
| 346 | X038 | China | Zhanggong, Jiangxi | *submitting* |
| 347 | X039 | China | Zhanggong, Jiangxi | *submitting* |
| 348 | X040 | China | Zhanggong, Jiangxi | *submitting* |
| 349 | X041 | China | Ruijing, Jiangxi | *submitting* |
| 350 | X042 | China | Ruijing, Jiangxi | *submitting* |
| 351 | X043 | China | Longnan, Jiangxi | *submitting* |
| 352 | X044 | China | Longnan, Jiangxi | *submitting* |
| 353 | X045 | China | Shicheng, Jiangxi | *submitting* |
| 354 | X046 | China | Xingguo, Jiangxi | *submitting* |
| 355 | X047 | China | Xingguo, Jiangxi | *submitting* |
| 356 | X048 | China | Chongyi B, Jiangxi | *submitting* |
